# Gene duplication and an accelerated evolutionary rate in 11S globulin genes are associated with higher protein synthesis in dicots as compared to monocots

**DOI:** 10.1186/1471-2148-12-15

**Published:** 2012-01-31

**Authors:** Chun Li, Meng Li, Jim M Dunwell, Yuan-Ming Zhang

**Affiliations:** 1 State Key Laboratory of Crop Genetics and Germplasm Enhancement, College of Agriculture, Nanjing Agricultural University, Nanjing 210095, P R China; 2 Henan Sesame Research Center, Henan Academy of Agricultural Sciences, Zhengzhou 450002, P R China; 3 School of Biological Sciences, University of Reading, Whiteknights, Reading RG6 6AS, UK

**Keywords:** 11S globulin, dicot, evolutionary rate, gene duplication, legumins, monocot, positive selection

## Abstract

**Background:**

Seed storage proteins are a major source of dietary protein, and the content of such proteins determines both the quantity and quality of crop yield. Significantly, examination of the protein content in the seeds of crop plants shows a distinct difference between monocots and dicots. Thus, it is expected that there are different evolutionary patterns in the genes underlying protein synthesis in the seeds of these two groups of plants.

**Results:**

Gene duplication, evolutionary rate and positive selection of a major gene family of seed storage proteins (the 11S globulin genes), were compared in dicots and monocots. The results, obtained from five species in each group, show more gene duplications, a higher evolutionary rate and positive selections of this gene family in dicots, which are rich in 11S globulins, but not in the monocots.

**Conclusion:**

Our findings provide evidence to support the suggestion that gene duplication and an accelerated evolutionary rate may be associated with higher protein synthesis in dicots as compared to monocots.

## Background

The plant seed is not only an organ of propagation and dispersal but also the major plant tissue harvested and used either directly as part of the human diet or as feed for animals. At the present time there is concern over long term food security and the impact of the move towards meat-based diets that will lead to a significant increase in the demand for plant protein for animal feed [1]. The amount of protein present in plant seeds varies from ~10% of the dry weight in most monocot (e.g. *O. sativa*, *S. bicolor*, *S. italica*, *Z. mays *and *B. distachyon*) to more than 30% in most dicots (e.g. *G. max*, *R. communis*, *C. sativus *and *A. thaliana*), and forms a major source of dietary protein [2-7]. To determine whether differences in evolutionary patterns may explain the phenotypic differences observed, a comparative investigation of evolutionary divergence in genes underlying protein synthesis in these two groups of plants is thus warranted.

Seed storage proteins can be classified into four groups: albumins, globulins, prolamins and glutelins [8]. Albumins and globulins comprise the storage proteins of dicots, whereas prolamins and glutelins are the major proteins in monocots [4,9,10]. 2S albumins, a major class of dicot seed storage proteins, have been most widely studied in the Cruciferae, notably *B. napus *and *A. thaliana *[9,10]. Prolamins, the major endosperm storage proteins of all cereal grains, with the exceptions of oats and rice, can be classified into many subgroups, e.g. sulphur-rich (S-rich), sulphur-poor (S-poor) and high molecular weight (HMW) prolamins [4]. The globulins, the most widely distributed group of storage proteins, are part of the cupin superfamily [11] and are evolved from bacterial enzymes. The globulins are present not only in dicots but also in monocots [9] and can be divided into 7S vicilin-type and 11S legumin-type globulins according to their sedimentation coefficients. It should be noted that the genes encoding the 11S-type globulins in monocots are the same gene family, 11S globulin family, as those in dicots; whereas the genes encoding the 7S-type globulins in monocots are not evolutionarily related to those in dicots [4]. Thus, we focus on the genes encoding the 11S-type globulins in this study.

Many efforts have been made to describe the gene families encoding seed storage proteins. For albumins, they are encoded by multi-gene families in many dicots (e.g., *A. thaliana *and *B. napus*); and evolutionary research into the gene families suggests that the albumin genes were duplicated prior to the Brassiceae-Sysimbrieae split, and gene duplication has played a role in their evolution [12,13]. For prolamins, they are the major seed storage proteins in most grass species (e.g., *Z. mays *and *S. bicolor*); and studies have suggested that the prolamin gene families have undergone many rounds of gene duplication [14,15]. For globulins, eight, four, eleven and fourteen gene have been identified and classified in *G. max*, *A. thaliana*, *R. communis *and *O. sativa*, respectively [16-24].

From the research described above, it can be concluded that the seed storage protein gene families have expanded in a lineage-specific manner through gene duplication. As a major process in the evolution, gene duplications can provide raw material for evolution by producing new copies. The human globin gene family is a representative example: several globin genes have arisen from a single ancestral precursor, thus making individual genes available to take on specialized roles, with some genes becoming active during embryonic and fetal development, and others becoming active in the adult organism [25]. Gene duplications may also affect phenotype by altering gene dosage: the amount of protein synthesized is often proportional to the number of gene copies present, so extra genes can lead to excess proteins. This applies to many kinds of genes, such as rRNAs, tRNA and histones [26,27]. A critical question thus can be asked: does gene duplication contribute to the higher levels of protein synthesis in dicots than in monocots? On the other hand, gene duplication usually brings variation in evolutionary rate [26], and such variation has been predicted to be associated with phenotypic differences. For example, Hunt et al. [28] investigated the evolution of genes associated with phenotypically plastic castes, sexes, and developmental stages of the fire ant *Solenopsis invicta*, and argued that an elevated rate is a precursor to the evolution of phenotypic differences. Thus, we are also interested in another question: does an accelerated evolutionary rate play a role in the evolution of storage protein content?

To shed light on the two questions above, we investigated the process of molecular evolution of the 11S globulin gene family, which is widely distributed in dicot and monocot species, by comparing the differing evolutionary patterns in the two groups. Our analyses suggested that gene duplication and an accelerated evolutionary rate in 11S globulin genes may be associated with higher protein synthesis in dicots than in monocots.

## Results

### Sequences retrieval and phylogenetic analysis

**Figure 1:**
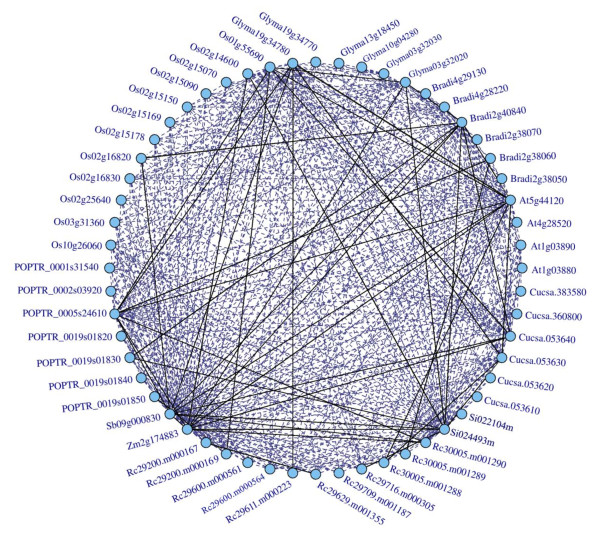
**The COG of 11S globulin gene family**. Solid lines show symmetrical BeTs (the Best Hits) and broken lines show asymmetrical BeTs. Genes from the same species are adjacent. Gene ID is indicated and the prefix "Rc" denotes IDs from *Ricinus communis*. Among these IDs, At1g03880, At1g03890, At4g28520 and At5g44120 are known to encode *CRB, CRU2, CRC* and *CRA1*, respectively; Glyma03g32030, Glyma03g32020, Glyma19g34780, Glyma10g04280, Glyma13g18450 and Glyma19g34770 to encode *Gy1-5 *and *Gy7*, respectively; Rc29600.m000561, Rc29600.m000564, Rc30005.m001289, Rc30005.m001290, Rc29611.m000223, Rc29200.m000169, Rc29629.m001355, Rc29709.m001187, Rc29716.m000305, Rc29200.m000167 and Rc30005.m001288 to encode *RcLEG1-1 *to *RcLEG1-5 *and *RcLEG2-1 *to *RcLEG2-6*, respectively; and Os01g55690, Os10g26060, Os03g31360, Os02g15169, Os02g15178, Os02g15150, Os02g16820, Os02g16830, Os02g14600, Os02g15070, Os02g25640 and Os02g15090 to encode *GluA-1*, *GluA-2*, *GluA-3, GluB-1a*, *GluB-1b*, *GluB-2*, *GluB-5*, *GluB-4*, *GluB-7*, *GluB-6*, *GluC-1 *and *GluD*, respectively.

We collect the sequences of 11S globulin genes through a COG method. This procedure is based a simple notion that, if any proteins from distant genomes are more similar to each other than to any other proteins from the same genomes, they are most likely to belong to an orthologous family. In such a family, there are two kinds of relationship between a pair of sequences, namely symmetrical and asymmetrical BeTs (the Best Hits). If the symmetrical and asymmetrical BeTs are linked respectively by solid and broken lines, the orthologous family would forms a network; and thus all other members in the network can be identified when one member was investigated [29]. The 11S globulin genes from the five dicots and the five monocots form a COG containing 56 sequences (Figure 1). Of these genes, four genes encoding 12S globulins [20] come from *A. thaliana*; six genes, *Gy1*-*Gy5 *and *Gy7 *[16], come from *G. max*; twelve genes, all the functional glutelin genes, come from *O. sativa *[24]; eleven genes described in [7] come from *R. communis*; and six, seven, six, one, one and two genes come from *C. sativus*, *P. trichocarpa, B. distachyon, Z. mays*, *S. bicolor *and *S. italica*, respectively.

**Figure 2:**
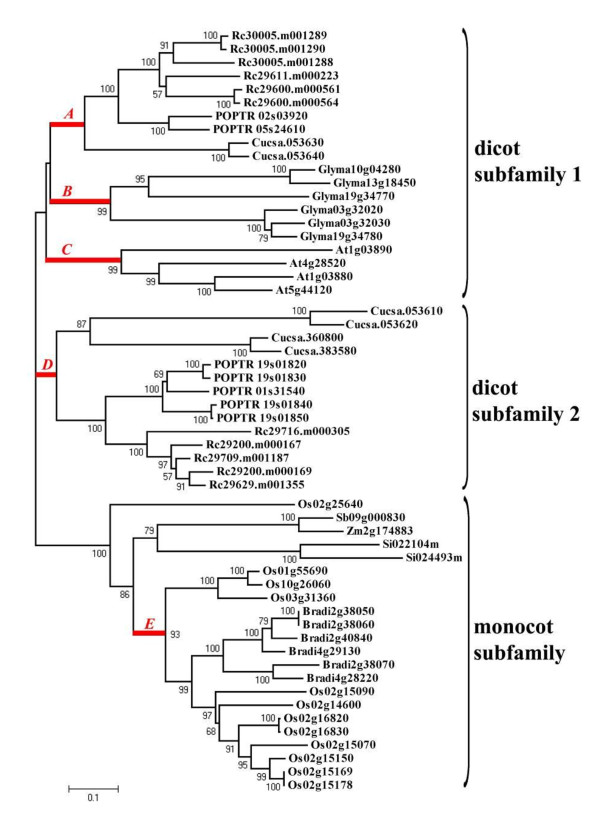
**Phylogenetic relationships of sequences within the 11S globulin gene family by neighbor joining (NJ) method with bootstrap support above 50% shown at the nodes**. Letter *A*-*E *indicates the branches used in analysis of evolutionary rate and positive selection.

In order to analyze the phylogenetic relationship of the 11S globulin genes from the above ten species, the NJ and Bayesian methods were used to reconstruct the phylogenetic tree. Similar results were achieved (data not shown), and the NJ tree is shown in Figure 2, in which there are three subfamily clades: dicot subfamily 1, dicot subfamily 2 and monocot subfamily. The dicot subfamily 1 contains all the genes from *A. thaliana *and *G. max*, two genes from *C. sativus*, two genes from *P. trichocarpa *and *RcLEG1 *of *R. communis*; the dicot subfamily 2 contains the other genes from *C. sativus*, *P. trichocarpa *and *R. communis*; and the monocot subfamily contains all the genes from the five monocot species. In the three subfamilies, with the exception of rice, genes from the same species form monophyletic groups; and the phylogenetic relationship of the monophyletic groups is largely concordant with the species tree described by [30].

### Gene duplication in 11S globulin gene family

In the 11S globulin gene family, there are four or more genes from *C. sativus*, *P. trichocarpa*, *R. communis*, *A. thaliana*, *G. max*, *B. distachyon *and *O. sativa*, suggesting that the 11S globulin genes in these species have undergone two or more rounds of duplication. Among these duplications, tandem duplication is a major type, and occurred in all the species above, e.g. Rc30005.m001289-Rc30005.m001290 and At1g03880-At1g03890 and Os02g16820-Os02g16830. Furthermore, most of the 11S globulin genes from the dicot species are located on the chromosome segments that share conserved synteny with each other [18], suggesting that segment duplication or whole genome duplication (WGD) is the major origin of duplicate genes.

### Evolutionary rate of 11S globulin gene family

To determine whether there were different evolutionary patterns across different subfamilies of the 11S globulin genes in monocots and dicots, the *ω *values for these genes were calculated by a branch-specific model.

In the branch-specific model, three cases were considered in this study. One is a two-ratio model that suggests there are distinct monocot (*ω*_0_) and dicot subfamilies (*ω*_1_), one is a three-ratio model that suggests there is one monocot (*ω*_0_) and two dicot subfamilies 1 (*ω*_1_) and 2 (*ω*_2_), and one is a six-ratio model that suggests there are subclades under the branches *A*-*E *(*ω*_1 _~ *ω*_5_) and the other (*ω*_0_). All the above models were favored over the one-ratio model by the likelihood ratio test (P < 0.05, Table 1) and in the two-, three- and six-ratio models, the *ω *estimate for the dicot subfamily is considerably higher than that for the monocot subfamily. These results suggest that the dicot 11S globulin genes are under reduced evolutionary constraints, and thus evolve at a higher evolutionary rate. We should point out that in the six-ratio model, the genes from *S. italica*, *Z. mays *and *S. bicolor *were not taken into account, because they may have a different evolutionary pattern due to the fact that 11S globulins are minor components [4,31] and there are only one or two 11S globulin gene copies in these species; and that branches *A*-*E *were chosen because five branches can be grouped for the phylogenetic tree.

**Table 1:** Evolutionary rate analysis of 11S globulin family using branch-specific model of PAML

Model	*ω *setting	-*ln *L	Estimated parameters	Likelihood ratio test
One-ratio	entire tree: *ω*_0_	38214.64	*ω*_0 _= 0.234	
Two-ratio	monocot subfamily: *ω*_0_	38210.14	*ω*_0 _= 0.212	two ratio *vs*. one ratio: *P *< 0.01
	dicot subfamily 1& 2: *ω*_1_		*ω*_1 _= 0.249	
Three-ratio	monocot subfamily: *ω*_0_	38209.98	*ω*_0 _= 0.212	three ratio *vs*. one ratio: *P *< 0.01
	dicot subfamily 1: *ω*_1_		*ω*_1 _= 0.252	three ratio *vs*. two ratio: *P *> 0.05
	dicot subfamily 2: *ω*_2_		*ω*_2 _= 0.243	
Six-ratio	branch *A*: *ω*_1_	38182.58	*ω*_1 _= 0.225	six ratio *vs*. one ratio: *P *< 0.01
	branch *B*: *ω*_2_		*ω*_2 _= 0.373	six ratio *vs*. two ratio: *P *< 0.01
	branch *C*: *ω*_3_		*ω*_3 _= 0.198	six ratio *vs*. three ratio: *P *< 0.01
	branch *D*: *ω*_4_		*ω*_4 _= 0.242	
	branch *E*: *ω*_5_		*ω*_5 _= 0.187	
	other branches: *ω*_0_		*ω*_0 _= 0.283	

### Positive selection in the 11S globulin genes

To test for positive selection in the 11S globulin gene family, branches *A*, *B*, *C*, *D *and *E *were independently defined as the foreground branch in the branch-site model (Figure 2). When branches *A*, *B*, *C *or *D *were defined as the foreground branch, the null hypothesis is rejected (*P <*0.01); and the estimated parameters of the alternative hypotheses indicate that about 5%-11% sites on these branches were under positive selection with a *ω *value (*ω*_2_) larger than one. When branch *E *was brought to the foreground, the null hypothesis cannot be rejected, and thus no significant positive selection was detected (Table 2). This result suggests that positive selection mainly occurred in the 11S globulin genes in dicots.

**Table 2:** Summary statistics for detecting selection using branch-site models of PAML

Foreground branch	Null hypothesis		Alternative hypothesis	
		
	-ln *L*	Estimated parameters	-ln *L*	Estimated parameters
**Branch *A***	46859.30	*p*_0 _= 0.66, *p*_1 _= 0.28 (*p*_2_+*p*_3 _= 0.06)	46851.6**	*p*_0 _= 0.69, *p*_1 _= 0.27 (*p*_2_+*p*_3 _= 0.04)
		*ω*_0 _= 0.23, *ω*_1 _= *ω*_2 _= 1		*ω*_0 _= 0.23, *ω*_1 _= 1.00, *ω*_2 _= 999
**Branch *B***	46854.00	*p*_0 _= 0.53, *p*_1 _= 0.22 (*p*_2_+*p*_3 _= 0.25)	46837.66**	*p*_0 _= 0.63, *p*_1 _= 0.26 (*p*_2_+*p*_3 _= 0.11)
		*ω*_0 _= 0.23, *ω*_1 _= *ω*_2 _= 1		*ω*_0 _= 0.23, *ω*_1 _= 1.00, *ω*_2 _= 56.08
**Branch *C***	46852.38	*p*_0 _= 0.53, *p*_1 _= 0.21 (*p*_2_+*p*_3 _= 0.36)	46831.76**	*p*_0 _= 0.64, *p*_1 _= 0.26 (*p*_2_+*p*_3 _= 0.10)
		*ω*_0 _= 0.23, *ω*_1 _= *ω*_2 _= 1		*ω*_0 _= 0.23, *ω*_1 _= 1.00, *ω*_2 _= 526.55
**Branch *D***	46859.11	*p*_0 _= 0.61, *p*_1 _= 0.26 (*p*_2_+*p*_3 _= 0.13)	46842.30**	*p*_0 _= 0.68, *p*_1 _= 0.27 (*p*_2_+*p*_3 _= 0.05)
		*ω*_0 _= 0.23, *ω*_1 _= *ω*_2 _= 1		*ω*_0 _= 0.23, *ω*_1 _= 1.00, *ω*_2 _= 999
**Branch *E***	46859.33	*p*_0 _= 0.70, *p*_1 _= 0.30 (*p*_2_+*p*_3 _< 0.01)	46859.33	*p*_0 _= 0.70, *p*_1 _= 0.30 (*p*_2_+*p*_3 _< 0.01)
		*ω*_0 _= 0.23, *ω*_1 _= *ω*_2 _= 1		*ω*_0 _= 0.23, *ω*_1 _= *ω*_2 _= 1

Note: ** P <0.01, calculated from LRT.

## Discussion and Conclusions

### Gene duplication may be associated with the higher 11S globulin content in dicots

In our duplication analyses, we found four or more 11S globulin genes in each of the five dicot species analyzed. It appears that higher number of duplicates is a feature of the dicot 11S globulin genes, rather than being randomly produced by the hitchhiking effect of genome duplication, because:

i) The copy number of the dicot 11S globulin gene is higher than the average copy number of the genome; for example, in the genome of *A. thaliana*, 80% of genes recovered to single copy through gene loss in a short period after the duplications, resulting an average of 2.3 copies per family [32], which is lower than the 4 duplicates in the 11S globulin gene family; in the genome of *G. max*, 74.1% and 56.6% genes were lost following the early and the recent WGD, respectively, resulting an average of about 3 copies per family [33,34], which is lower than the 6 duplicates in the globulin gene family;

ii) In each of the five dicots, there are 11S globulin genes that arose from tandem duplications; and

iii) The genomes of *S. italica*, *Z. mays *and *S. bicolor *are thought to have undergone several rounds of duplications [35,36], but they contain only one or two 11S globulin genes, suggesting that gene losses are common in the family, and thus implying that the duplicates of the 11S globulin genes are preferentially retained in the dicot species.

We hypothesize that the higher number of duplicates may be associated with higher 11S globulin content in dicots. The reasons are as follows.

First, the seed 11S globulin content in the species with a higher copy number of 11S globulin genes is greater than that in those with a lower copy number (Table 3). In the dicot species *A. thaliana*, *R. communis*, *G. max *and *C. sativus*, the copy number of 11S globulin genes is four or more, and the 11S globulins are predominant among the seed storage proteins [2,5,20,37-41]. In the monocot species *S. italica*, *Z. mays *and *S. bicolor*, the copy number of 11S globulin genes is one or two, and 11S globulins are minor [4,31]. However, there are two exceptions, *O. sativa *and *B. distachyon *(monocots). In these two species, the copy number of 11S globulin genes is six or more and the 11S globulins are predominant among the seed storage proteins [6,42-44], although the overall protein content is low.

**Table 3:** The content of seed 11S globulins and the copy number of their genes

Group	Species	Seed storage protein^1^	11S globulins^2^	No. of genes
dicot	*Arabidopsis thaliana*	30-40%	major component	4
	*Glycine max*	~40%	~40%	6
	*Cucumis sativus*	~35%	major component	6
	*Populus trichocarpa*	major component	unknown	7
	*Ricinus communis*	~40%	~75%	11
monocot	*Brachypodium distachyon*	< 10%	~60%	6
	*Oryza sativa*	< 10%	~70%	12
	*Setaria italica*	~10%	minor component	2
	*Zea mays*	~7%	minor component	1
	*Sorghum bicolor*	< 10%	minor component	1

Note: ^1 ^percentage to the seed dry weight; ^2 ^percentage to the seed storage protein. The references are listed in the main text.

Second, the presence of duplicate genes leads to the production of an extra amount of protein, because extra mRNA can be produced [26]. There is some evidence for this viewpoint: i) the absence of, or preferential expression of, the 11S globulin genes in *G. max *leads to glycinin deficiency or greater accumulation, respectively [45,46]; ii) of the glycinin gene groups I (*Gy1*-*Gy3*), IIa (*Gy4*) and IIb (*Gy5*) in *G. max*, a mutation with the absence of one or two groups of genes leads to a decrease in glycinin content [47]; and simultaneous mutation of all the genes leads to the lack of all the glycinin polypeptides [48]. Third, enlarging the 2S albumin gene family of *A. thaliana *by introducing an extra member leads to an increase in transcript production [49].

Finally, in the investigation of ancestral whole genome duplication in seed plants and angiosperms, Jiao *et al. *[50] proposed that there were two WGDs in ancestral lineages shortly before the diversification of extant seed plants and extant angiosperms respectively, and argued that these ancestral WGDs i) resulted in the diversification of regulatory genes important to seed and flower development; ii) were involved in major innovations that ultimately contributed to the rise and eventual dominance of seed plants and angiosperms; and iii) enabled flowering plants to enjoy a distinct evolutionary advantage that allowed them to survive harsh climatic changes and even mass extinctions. The seed storage proteins provide essential nutrition for seed germination and development, and thus are vital for species survival and adaptation. Therefore, the genes governing the seed storage proteins are expected to have a higher number of duplications, leading to improved phenotypic robustness and an evolutionary advantage.

### Higher evolutionary rate may be associated with the higher 11S globulin content in dicots

In the evolutionary rate analyses, a consistent conclusion, that the dicot 11S globulin genes evolve more rapidly, was achieved from the three branch-specific models, i.e. two-, three- and six-ratio models (Table 1). We hypothesize that accelerated evolutionary rate may also be associated with the higher 11S globulin content in dicots. The reasons are as follows.

Positive selection leads to an accelerated evolutionary rate of the 11S globulin genes in dicots, and may also lead to a higher ability of dicots to produce 11S globulins. Positive selection is a major factor affecting the evolutionary rate of a gene [51]. To investigate evidence for positive selection in the 11S globulin genes, we analyzed five branches, i.e. branches *A*-*E *(Figure 2), which represent the major origin events in the evolution of the 11S globulin gene families, e.g. branch *B *represents the origin of *G. max *11S globulin gene family. Of these branches, branches *A*-*D*, being the dicot 11S globulin genes, were proved to have undergone positive selection, whereas branch *E*, being the monocot 11S globulin genes, did not. This result provides evidence that positive selection may lead to a higher ability of dicots to produce 11S globulins.

To shed more light on the role of gene duplication and accelerated rates of evolution in producing the observed patterns of divergence in protein synthesis between dicots and monocots, future studies investigating other types of seed storage proteins and a broader range of plant species will be needed.

## Methods

### Sequences Retrieval and Comparisons

Sequences retrieval and comparisons were performed using the method described by Tatusov *et al. *[29] with a slight modification. Briefly, our method included the following steps:

1) Amino acid sequences of proteins were downloaded from JGI (http://www.phytozome.net/), the maize sequence from http://www.maizesequence.org, and used to construct a local BLAST database using BLAST 2.2.24. The species are listed in Table 4.

**Table 4:** Source of the 11S globulin genes used in this study

Group	Common name	Species name	Version	No of genes	Reference
dicot	thale cress	*Arabidopsis thaliana*	TAIR 9	33,410	[62]
	soybean	*Glycine max*	1.0	75,778	[34]
	cucumber	*Cucumis sativus*	122	32,509	[63]
	black cottonwood	*Populus trichocarpa*	2.0	45,778	[64]
	castor bean	*Ricinus communis*	0.1	31,221	[65]
monocot	purple false brome	*Brachypodium distachyon*	1.0	32,255	[66]
	rice	*Oryza sativa*	MSU 6.0	67,393	[67]
	foxtail millet	*Setaria italica*	2.1	38,038	
	maize	*Zea mays*	5a	53,764	[68]
	sorghum	*Sorghum bicolor*	1.0	36,338	[69]

2) An all-against-all protein sequence comparison was carried out.

3) In all the comparisons produced in the step 2, the ones with *G. max Gy1 *[16] as a query were identified, and the obvious paralogs were collapsed.

4) All interspecies Best Hits (BeTs) of *Gy1 *and their paralogs were detected.

5) Steps 3) and 4) were repeated, with the resulting sequences as secondary BLASTp queries until no new sequence was found.

6) The protocol of Tatusov *et al. *[29] was applied to all the sequences from the analysis above and used to form a Clusters of Orthologous Groups (COG).

7) A case-by-case analysis of the COG was conducted. This analysis served to eliminate false-positives and to ensure all homologs were included.

This approach was based on the consistency between genome-specific best hits, rather than the absolute level of similarity; it therefore allows the detection of orthologs among both slowly and quickly evolving genes.

### Phylogenetic Analyses

The cDNA sequences were aligned using the codon model of program PRANK (100701 version) using the default options [52], and were then translated into amino acid sequences. Phylogenetic tree reconstruction was carried out using both Neighbor-Joining (NJ) and Bayesian approaches based on the aligned amino acid sequences. In the NJ method, the phylogenetic analyses were conducted using the MEGA 5 program [53]. The parameter setups were as follows: model, Jones-Taylor-Thornton (JTT) [54]; bootstrap, 1000 replicates; and gap/missing data, pairwise-deletion.

In the Bayesian method, the analyses were conducted using MrBayes v3.1 [55]. The parameter setups were as follows: JTT substitution model, four chains, one million generations, two runs, sampling every 100 generations and discarding a burn-in of 250,000 generations.

### Estimation of d_N_/d_S _Ratios

The coding sequences were aligned using the codon model of PRANK software (100701 version) using the default options [52], and alignment gaps were deleted manually.

On the basis of the aligned coding sequences, the pairwise ratio of non-synonymous substitutions per non-synonymous site (*d*_N_) to the synonymous substitutions per synonymous site (*d*_S_) (*ω *value) of homologous genes was calculated by the maximum likelihood method in Codeml from the PAML package v4.4 [56]. Saturation effects were avoided by discarding the gene pairs for which *d*_S _> 2 [57].

The branch-specific model, which allows the *ω *ratio to vary among the branches in the phylogeny, was used to test whether there are different *ω *values on particular lineages [58]. If the *ω *ratio among all the branches is a constant, the model can be changed into the one-ratio model. Thus the likelihood ratio test (LRT) was used to test whether the data fit the branch-specific model significantly better than the one-ratio model [58].

### Detection of Positive Selection

The aligned codon sequences were used to test positive selection using the branch-site model implemented in the program Codeml of PAML 4.4 [56]. This model allows *ω *to vary both among sites in the sequences and across branches on the tree and its purpose is to detect positive selection affecting a few sites along particular lineages (called foreground branches). The model assumes that there are four site classes in the sequence. The first class of sites is highly conserved in all lineages with a small *ω *ratio, *ω*_0_. The second class includes neutral or weakly constrained sites for which *ω *= *ω*_1_, where *ω*_1 _is near or smaller than 1. In the third and fourth classes, the background lineages show *ω*_0 _or *ω*_1_, but foreground branches have *ω*_2_, which may be greater than 1. In the LRT, the null hypothesis fixes *ω*_2 _= 1 (neutral selection) and the alternative hypothesis constrains *ω*_2 _≥ 1 (positive selection) [59,60]. In the existence of positive selection, the posterior probabilities for the sites with positive selection were calculated by the Bayes empirical Bayes method (BEB) [61].

## Abbreviations

BEB: the Bayes empirical Bayes; BeTs: Best Hits; COG: Clusters of Orthologous Groups; HMW: high molecular weight; JTT: Jones-Taylor-Thornton; LRT: likelihood ratio test; NJ: Neighbor Joining; WGD: whole genome duplication

## Authors' contributions

YMZ designed the study, coordinated and supervised the analysis. CL and ML performed the analysis and drafted the paper. YMZ and JMD revised the manuscript. All authors read and approved the final manuscript.
